# The Impact of Vitamin K2 (Menaquionones) in Children’s Health and Diseases: A Review of the Literature

**DOI:** 10.3390/children9010078

**Published:** 2022-01-05

**Authors:** Agnieszka Kozioł-Kozakowska, Katarzyna Maresz

**Affiliations:** 1 Department of Pediatrics, Gastroenterology and Nutrition, Institute of Pediatrics, Faculty of Medicine, Jagiellonian University Medical College, 30-663 Kraków, Poland; 2 International Science & Health Foundation, 30-134 Kraków, Poland; katarzyna.maresz@nutricon.eu

**Keywords:** vitamin K, vitamin K2, menaquinone, vitamin K-dependent proteins, vitamin K deficiency bleeding, breast milk, fractures, childhood illnesses

## Abstract

Vitamin K2 activates vitamin K-dependent proteins that support many biological functions, such as bone mineralization, the inhibition of vascular stiffness, the improvement of endothelial function, the maintenance of strong teeth, brain development, joint health, and optimal body weight. Due to the transformation of food habits in developed countries over the last five decades, vitamin K and, specifically, vitamin K2 intakes among parents and their offspring have decreased significantly, resulting in serious health implications. The therapeutics used in pediatric practice (antibiotics and glucocorticoids) are also to blame for this situation. Low vitamin K status is much more frequent in newborns, due to both endogenous and exogenous insufficiencies. Just after birth vitamin K stores are low, and since human milk is relatively poor in this nutrient, breast-fed infants are at particular risk of a bleeding disorder called vitamin K deficiency bleeding. A pilot study showed that better vitamin K status is associated with lower rate of low-energy fracture incidence. An ongoing clinical trial is intended to address whether vitamin K2 and D3 supplementation might positively impact the biological process of bone healing. Vitamin K2 as menaquinone-7 (MK-7) has a documented history of safe and effective use. The lack of adverse effects of MK-7 makes it the ideal choice for supplementation by pregnant and nursing women and children, both healthy and suffering from various malabsorptions and health disorders, such as dyslipidemia, diabetes, thalassemia major (TM), cystic fibrosis (CF), inflammatory bowel diseases (IBD), and chronic liver diseases. Additionally, worthy of consideration is the use of vitamin K2 in obesity-related health outcomes.

## 1. Introduction

Vitamin K1 and K2 are fat-soluble vitamins. Vitamin K1 is found mainly in leafy green vegetables. Vitamin K2 (menaquinones) contains an unsaturated aliphatic side chain with a variable number of prenyl units. The number of prenyl units indicates the respective type of menaquinone. For example, MK-4 contains four prenyl units and MK-7 contains seven prenyl units. Vitamin K2 is mostly produced by bacteria, except for MK-4, which can be developed by tissue-specific conversion from vitamin K1 in animals. A traditional Japanese dish called “natto,” consisting of fermented soybeans, holds the highest content of K2, particularly MK-7.

Both vitamins K1 and K2 act as essential cofactors for the enzyme *gamma-glutamylcarboxylase* (GGCX) and in the carboxylation process of vitamin K-dependent proteins (VKDPs) [1,2,3]. Vitamin K2 has gained more appreciation in the recent years due to its longer half-life, better bioavailability, and extrahepatic activity in comparison with vitamin K1.

In addition to participating in the activation of VKDPs, vitamin K has been reported to operate as an anti-inflammatory and antioxidant agent independent of its GGCX cofactor activity [4,5]. A recent study, the objective of which was to estimate the effect of vitamin K on the redox metabolism, demonstrated that vitamin K prevented a redox imbalance by lowering reactive oxygen species (ROS) levels. The greatest effect was achieved with MK-7 [6].

Vitamin K2 is also a transcriptional regulator of bone-specific genes that functions through the steroid and xenobiotic receptor (SXR) to facilitate the expression of osteoblastic markers [7]. Moreover, vitamin K2 serves as a mitochondrial electron carrier, helping to maintain normal adenosine triphosphate (ATP) production [8]. The function of vitamin K2 was found to be much broader than K1 (Table 1).

The role of VKDPs in processes beyond coagulation has been discovered and well demonstrated in the past few decades. The evidence from numerous clinical trials clearly supports the benefit of high vitamin K2 consumption for human health. Vitamin K2 meets all criteria for a bioactive substance to be considered for a recommended daily intake (RDI). Many countries have an RDI for vitamin K1 based on earlier research. As vitamin K2 is very important for extrahepatic tissues, the recommendations should combine vitamin K1 and K2 intakes. The procedures by which vitamin K2 levels can be assessed call for standardization, and an RDI for vitamin K2 based on current research needs to be established and accepted worldwide.

The objective of this paper was to perform a systematic review of research papers. We also evaluated clinical trials that examined the role of vitamin K2 for children’s health and whether vitamin K2 supplementation influences markers of bone disease such as bone mineral density.

## 2. The Beneficial Role of Vitamin K2 in Physiological Processes

**Figure 1:**
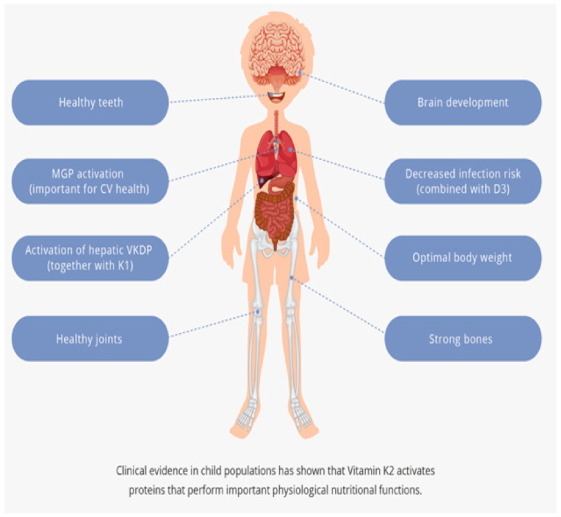
Vitamin K2 is important for many biological functions.

Vitamin K2 activates proteins performing crucial biological functions that range from bone mineralization and healthy teeth, through promoting cardiovascular health, to maintaining brain development, joint health, and optimal body weight (Figure 1).

### 2.1. Strong Bones

Osteocalcin is a VKDP synthesized by osteoblasts and is thought to be related to bone mineralization. Vitamin K is necessary for the activation of osteocalcin. Moreover, vitamin K2 has been shown to inhibit resorption of the bone by suppression of the prostaglandin E2 synthesis in osteoclasts [9]. Children have the highest needs for K vitamins, since bone formation and development are most intense during childhood and adolescence. The higher the bone mass acquired before the age of 20–25, the better prognosis for good bone health later in life, as bone mass declines throughout adulthood. By the age of 18 or 19, approximately 90% of peak bone mass is attained, with about 25% being acquired during the 2-year period surrounding peak height velocity of building bone mass [10,11]. The researchers showed that a 10% improvement in peak bone mass is correlated with the reduction of the risk of osteoporotic fracture in adulthood by half [12].

### 2.2. Healthy Teeth

According to the traditional view, dental caries are considered a tooth de-mineralizing process that takes place solely in the oral cavity. Attributed to a new perception of oral/systemic links is the view that dental caries are an uncontrolled inflammatory response regulated by the brain and mitigated through the hypothalamus/parotid axis of the endocrine system. Tooth vulnerability or resistance is determined by a signaling factor constituted by the role of free radicals in the hypothalamus. This systemic caries paradigm puts nutrition at the forefront of prophylaxis as it concentrates on the root of the disease rather than traditional symptom-focused prevention efforts [13].

Vitamin K2 works in concert with calcium and vitamin D and appears to have a significant antioxidant role. Therefore, vitamin K2 can help to considerably reduce tooth decay while also appearing to play a prospective saliva-buffering role in the exocrine parotid and other salivary glands [14].

### 2.3. Cardiovascular Health

While cardiovascular incidents are rare in children, clinical observations show that arterial calcification and atherosclerosis is a progressive process that accumulates over decades [15,16]. Research has demonstrated that the beginnings of arterial calcification have been observed in otherwise healthy children. Additionally, in children with auto-inflammatory conditions and children with kidney disorders a deficiency of vitamin K resulting in undercarboxylated non-functional matrix-gla protein (MGP) might contribute to the atherogenic process. For some children, this may begin as early as few years of age [17]. Results of many epidemiological studies showed that vitamin K2 is cardioprotective, and vitamin K1 is not. An optimal vitamin K2 intake is, therefore, important to support cardiovascular health [18,19,20].

### 2.4. Brain Development

Solid evidence exists that vitamin K2 plays important roles in the nervous system. Vitamin K2 is necessary to activate VKDPs in the brain, such as growth arrest-specific gene 6 protein (Gas6) and protein S, which are connected to cognitive processes. Gas6 and protein S influence different procedures in the brain, such as apoptosis, cell growth, and myelination [21]. Moreover, protein S is anticoagulant factor with direct cellular activities.

In the brain, vitamin K2 occurs mainly as MK-4. The highest concentrations of MK-4 were found in midbrain and pons medulla, and the lowest concentrations were observed in the cerebellum, olfactory bulb, thalamus, hippocampus, and striatum. Although MK-4 is the primary form in the brain, it was found that the supplementation of MK-7 increases the level of MK-4 in brain tissue [22].

Vitamin K is also involved in the synthesis of sphingolipids, crucial for the development of the functional integrity of the nervous system. Animal studies speak in favor of vitamin K2′s role in the biosynthesis of sphingolipids. Initially recognized for their role as building blocks of cell membranes, sphingolipids are now known to be involved in cell signaling, division, differentiation, and apoptosis. There are some studies, which showed the connection between alterations in sphingolipid metabolism and cognitive decline as well as neurodegenerative disorders such as Alzheimer’s and Parkinson’s diseases [23].

Moreover, animal and human studies suggest that optimal vitamin K status is important for psychomotor behavior and cognition. Additionally, in one report, vitamin K deficiency, due to administration of warfarin treatment during pregnancy, was associated with warfarin embryopathy. Some infants exhibited optic atrophy, developmental delay, and seizures after treating pregnant mothers with warfarin [24].

### 2.5. Activation of VKDP in the Liver

Vitamins K1 and K2 both activate VKDPs in the liver [25]. The deficiency of vitamin K in newborns might lead to life-threatening bleeding disorder of early infancy, namely vitamin K deficiency bleeding (VKDB), and potentially end in death of the neonate. There are different recommendations for vitamin K intake in different countries, but a consistent conclusion emerges: that optimal vitamin K status is important to support homeostasis processes in the body. Although in the majority of countries vitamin K1 is used to combat VKDB, we might find information that vitamin K2 as MK-4 has been used in addition in Japan [26].

### 2.6. Joint Health

Warfarin during pregnancy might impact newborn health. The resulting abnormalities include the cartilage and joints [24], which is why warfarin is contraindicated during, at least, the first three months of pregnancy [27].

Therefore, the optimal level of vitamin K is important for joint development. Moreover, it has been shown that VKDPs, inclusive of the mineralization inhibitor MGP, are detected in joint tissues, including cartilage and bone. It has been previously indicated that low vitamin K status is correlated with higher risk of osteoarthritis (OA) [28].

In human OA cartilage, MGP was found to be mainly inactive. Conversely, MGP is primarily carboxylated in healthy articular cartilage, suggesting the carboxylation of MGP is associated with OA [29].

### 2.7. Anti-Infection with D3

Respiratory tract infections (RTIs) are common in children, and vitamin D deficiency was shown to be associated with increased risk of RTI. Thus, vitamin D3 supplementation was accepted by medical community as an important nutrient, which reduces the risk of respiratory problems in children. It is, however, important to remember that vitamin D3 should be supplemented with vitamin K2 [30].

Currently, highest international interest is concentrated on the COVID-19 pandemic and potential ways of reducing its mortality rate. Researchers have recently shown that optimizing blood vitamin D and K levels could offer a solution for the reduction of mortality rate. Researchers proved the synergistic interplay between vitamins D and K on bone and cardiovascular health. Moreover, some scientists suggest that supplementation with vitamin D3 can be considered safe when it is combined with vitamin K2. New randomized controlled trials are needed to prove the discussed evidence [31].

## 3. The Importance of Vitamin K2 Supplementation in Various Pathological Conditions

### 3.1. Bone Fractures

During adolescence, the incidence of forearm fractures in children reaches its highest point as exercise increases. At the same time, cortical bone mass decreases due to increased calcium requirements in skeletal growth.

Physical activity and adequate nutrient intake have favorable influence on the bone quality. Hungarian researchers demonstrated that changes in bone mineral status among 10–12-year-old children, assessed by ultrasound method, are correlated with the amount of intense physical activity as well as with optimal vitamin K intake [32].

Vitamin K, as the γ-carboxylase cofactor, takes part in bone metabolism. Childrens’ requirements for this vitamin are among the greatest; its deficiency may have an impact on the peak bone mass formation, as well as on osteoporosis risk in adulthood.

The beneficial role of vitamin K, especially vitamin K2, in bone health is well established scientifically, based on epidemiological as well as on interventional studies published over the past decade. Of particular significance is the comprehensive attitude to bone health in children of all ages that includes optimal intake of calcium, vitamins D and K, balanced diet, and exercise. It has been shown that subclinical vitamin K and D deficiency is present in healthy pediatric population with low-energy bone fractures [33].

Further, a pilot study, which enrolled 20 children with radiologically confirmed low-energy fractures and 19 healthy children as a control group, showed that low vitamin K status, represented by the percentage of the active form of osteocalcin (UCR), correlates statistically with the low-energy fracture risk [34].

Based on a previous study, researchers assessed the impact of vitamin K2 on fracture repair. This clinical trial was accepted by the ethical committee and is ongoing. What has been learned so far is that low vitamin K status increases the fracture risk in kids. At this stage the positive effect of vitamin K2 on bone healing is only a hypothesis, but researchers are looking forward to the future results that will give us the answer whether vitamin K2 and D3 supplementation might positively impact this biological process [35].

### 3.2. Optimal Body Weight

Several authors have linked vitamin K status to fat and glucose metabolism. What lies behind obesity in childhood and adolescence is the development of insulin resistance that causes metabolic, structural, and functional changes, which may lead to increased risk of cardiovascular diseases and type 2 diabetes. Adolescence is also a crucial period when the so-called ‘adiposity rebound’ may occur, which means that adiposity increases after its lowest point in childhood. Children with a higher childhood BMI tend towards having a higher BMI, waist, and hip circumference in adulthood [36].

The results of a three-month animal study demonstrated that mice supplemented with vitamin K2 on the top of a high-fat diet gained less weight, less body fat, and showed decreased serum glucose and leptin in comparison with the control group on a high-fat diet only [37]. In humans, with high vitamin K2 intake and high vitamin K status, lower prevalence of metabolic syndrome was noted, and similar correlation was not found in case of vitamin K1. Moreover, better vitamin K status correlated with lower BMI [38].

So far, few studies have investigated the relationship between body weight and vitamin K intake. Nonetheless, it is acknowledged that suitable micronutrient intake is crucial to maintain different metabolic functions of the body, while inadequate intake is one of the top 10 risk components for the overall sickness burden globally [39]. These observations together with the outcome of new clinical trials in children, namely Vita-K ‘n’ Kids Study I & II (Vitamin K and Glucose Metabolism in Children at Risk for Diabetes & Vitamin K to Slow Progression of Dyslipidemia and Diabetes Risk), might bring us more useful information, soon, concerning the innovative roles for vitamin K in obesity-related health outcomes [40].

### 3.3. Cooley’s Anemia

Thalassemia major (TM) is also called Cooley’s anemia. People suffering from this most dangerous form of beta thalassemia have severe symptoms and life-threatening anemia. Their hemoglobin does not produce enough beta protein; therefore, they need regular blood transfusions and other medical treatment. Thalassemic osteopathy (TOSP) represents a salient factor determining risk for morbidity in patients with Cooley’s anemia and manifests as osteopenia/osteoporosis. A pilot study conducted among children with TM clearly showed that vitamin K2 and calcitriol combination positively affects bone mineral density (BMD). The authors suggested that patients with TOSP might benefit from K2 supplementation, although more clinical trials are needed to study the effects of treatment [41].

### 3.4. Cystic Fibrosis

People with cystic fibrosis (CF) may develop fat malabsorption due to pancreatic insufficiency, and this may also be linked to deficiencies of fat-soluble vitamins like vitamin K. Studies show that vitamin K deficiency is common in CF infants and toddlers and can even be detected in children receiving recommended supplementation. Moreover, a strong association between vitamin K status and clinical outcome in CF patients was found. Vitamin K intake was found to be an independent predictor of osteoblastic activity markers like: Glu-OC, Gla-OC, PICP (carboxy-terminal propeptide of type I procollagen), and PINP (serum amino-terminal propeptide of type I procollagen). Supplementation with vitamin K2 appears to be a good solution to improve the level of vitamin K in children with CF, but agreement needs to be reached on the appropriate dose and frequency of use of this nutrient [42,43,44].

### 3.5. Inflammatory Bowel Diseases (IBD)

Vitamin K deficiency was frequent in pediatric IBD patients, and more common in Crohn’s Disease (CD) than ulcerative colitis (UC) in pediatric patients [45]. There is a positive correlation between vitamin K deficiency and disease activity. In CD pediatric patients, vitamin K deficiency was more common in patients with higher CD activity [46,47]. In patients in clinical remission, vitamin K2 and vitamin D3 deficiencies were lower in the IBD group than in the control group, which may be affected by nutritional treatment and vitamin supplementation during the acute phase. It is suggested that vitamin K, along with vitamin D, are involved in the inhibition of inflammation and severity of the disease [48,49]. Animal study showed that vitamin K may act as an antioxidant and has anti-inflammatory functions. Vitamin K was proposed by the authors as a potential therapeutic nutrient in preventing oxidative damage and inhibiting inflammation in patients with ulcerative colitis [50]. Taking this assumption into account, vitamin K deficiency should lead to a more severe outcome of IBD, and its improved status may affect the course of the disease.

The reason for low vitamin K status in IBD remains unknown. It has been suggested that vitamin K deficiency is an effect of malabsorption caused by IBD, as well as by current treatment.

This is confirmed by the fact of the simultaneous deficiency of vitamin E in IBD and vitamin K metabolism disorders caused by antibiotics [51,52,53]. It is also likely that the conversion of K1 to the more effective menaquinones (K2) in people with inflammation and intestinal dysbiosis is altered [54].

In addition to the disease mechanism itself, the diet plays a key role in the development of vitamin K deficiency. Patients suffering from IBDs frequently consume inadequate amounts of the minerals and vitamins, resulting in nutritional deficiencies [55,56]. Good food sources of vitamin K are green vegetables and fermented dairy products, and, in one study, the consumption of these good sources of vitamin K was low in IBD patients’ diets. Children did not eat these foods every day nor were recommended nutritional norms for vitamin K. Improper diet affects not only the deficiency of vitamin K, but also the intestinal microbiota. Low intake of fruits and vegetables rich in fiber can change the profile of microbiota, which increases the risk of developing IBD, as well as influences the course of the disease [57].

Pediatric IBD patients seem to be prone to vitamin K deficiency due to ongoing inflammation. Consistent with the effects of vitamin K on bone metabolism, numerous clinical studies in adults showed that low vitamin K status contributes to low BMD in CD patients [58,59]. Clinical studies also demonstrated that vitamin K2 supplementation improves extrahepatic vitamin K status [60]. However, the supplementation with vitamin K2 for 12 months was not sufficient to boost bone mass. Nonetheless, the results showed that K2 supplementation improved vitamin K status represented as the percentage of active form of osteocalcin [61]. The potential impact of vitamins D and K to preserve bone health in IBD may unfortunately be alleviated by other circumstances such as the treatment with steroids or limited exposure to sunlight. There were no studies in the pediatric IBD population related to vitamin K treatment.

### 3.6. Liver Diseases

Due to malabsorption of fats and an inadequate diet, children with chronic liver disease are exposed to ongoing vitamin K deficiency, which increases the risk of hemorrhages and fractures and is related to degree of cholestasis and severity of liver disease. Low vitamin K status was shown to be common in children with mild to moderate chronic cholestatic liver disease, even despite vitamin K supplementation [62,63]. Adequate vitamin K level in children with chronic liver disease is crucial not only for the coagulation but also bone formation, and healthy development. The appropriate strategy for improvement of vitamin K status in young patients with chronic cholestatic liver disease should be established, because current recommendations seem not to be optimal, in our opinion.

### 3.7. Severe Disability

The researchers in Japan found that severely disabled children suffer from deficiencies of various nutritional compounds. There are many factors that contribute to low vitamin K status such as malnutrition, malabsorption, changes in microbiota, and also hepatic dysfunction.

The researchers showed that more than 40% of severely disabled children have vitamin K deficiency. In the group with vitamin deficiency, the majority of children demonstrated a bleeding tendency, more than 60% developed vitamin K deficiency in association with infection, and over 40% of them were treated with antibiotics. The good news is that all showed a good response to vitamin K2 supplementation, which seems to be especially important for disabled children in case of long-term antibiotic treatment [64].

## 4. The Negative Impact of Prolonged/Chronic Medication on Vitamin K Status

### 4.1. Long-Term Antibiotic Use

A significant number of pediatric patient visits each year end with the prescription of antibacterial drugs. Antibiotic therapy affects vitamin K levels in children. It has been shown that there are changes in gut microbiota due to antibiotics that alter intestinal vitamin K production. The level of K2 in the liver is lowered in people on antibiotics, especially on the cephalosporins, due to impairment the recycling of vitamin K.

Vitamin K deficiency is known to cause coagulopathy and bleeding in children on prolonged antibiotic treatment, therefore, in seriously ill patients on extended periods of antimicrobial drugs and inadequate diet, vitamin K2 prophylaxis is suggested to prevent morbidity and mortality [52,65,66].

### 4.2. Long-Term Glucocorticoid Use

For children who suffer from chronic diseases, the immunosuppressive and anti-inflammatory properties of oral corticosteroids constitute an inevitable treatment option. Their prolonged use may lead to various adverse drug reactions (ADRs), such as significant reductions in bone formation through the inhibition of osteoblasts. Moreover, corticosteroid use increases calcium excretion in the urine. Therefore, osteoporosis is considered a particular complication of chronic childhood illnesses cured with glucocorticoids (GCs) [67,68].

The adverse effects of GCs on bone formation are more pronounced in the growing skeleton, where trabecular and cortical bone growth is negatively affected. Decreased bone quality has been found in different disorders that require GCs, and a clinical study reported increased fracture risk in children who require more than four courses of GCs [69].

Japanese researchers found that vitamin K2 drugs might be effective to prevent bone fracture in glucocorticoid-induced osteoporosis (GIOP), because they increase bone strength independently of BMD [70].

Th authors of a systematic review, who critically evaluated the treatment options used in the management of bone loss associated with GC use among children, established that vitamin K2 (menatetrenone) combined with alfacalcidol has a beneficial effect on BMD in children who were treated with GCs for a longer time [71].

It was also reported by a team of Japanese researchers that treatment with alfacalcidol and vitamin K2 (as MK4) is beneficial for supporting bone health in children with skeletal unloading. The results of this pilot study showed that vitamin K2 as MK4 effectively and safely improves lumbar BMD in long-term prednisolone-treated children [72].

Vitamin K2 treatment has been shown to decrease the osteoporotic bone loss including GIOP [73]. It is an important nutrient, which supports strong bones and prevents bone detriment in patients on GCs therapy.

## 5. Children Have the Highest Needs for Vitamin K

In recent decades, vitamin K2 (menaquinone) has been specifically highlighted as a crucial cardiovascular and bone health nutrient [74].

Since the 1950s, many children and adults get less of this important nutrient than they should, and substantially lower vitamin K intakes may have serious health implications. Two cohorts of 4-year-old children were compared. A cohort born in the 1950s was compared in terms of dietary intake and sources of vitamin K with children born in the 1990s and showed that dietary vitamin K intake was significantly higher in the 1950s (39 mcg/day) than in the 1990s (24 mcg/day). Between the 1950s and the 1990s, people started to consume less vegetables and more fats and oils, therefore, sources of vitamin K intake have changed remarkably. Due to general changes in food habits (i.e., emphasizing fast snacks and processed meals), vitamin K intakes of children on Western diets have been on a significant decline since 1950s [75].

During the sharp increase in pubescence children seem to be extremely exposed to forearm fractures. Factors responsible for this regularity are closely related to each other and include skeletal growth, elevated need for calcium, increased physical activity, and decreased cortical bone mass. In recent years researchers aimed to check whether and how this regularity has changed. The outcome of a population-based study that was conducted in Minnesota over four time periods, 1969–1971, 1979–1981, 1989–1991, and 1999–2001, demonstrated that yearly incidence rates of forearm fractures per 100,000 escalated from 263.3 in 1969–1971 to 322.3 in 1979–1981, and to 399.8 in 1989–1991 before stabilizing at 372.9 in 1999–2001. Age-adjusted incidence rates per 100,000 were 32% higher among males in 1999–2001 compared with 1969–1971, and 56% greater among females in the same time periods [76].

**Figure 2:**
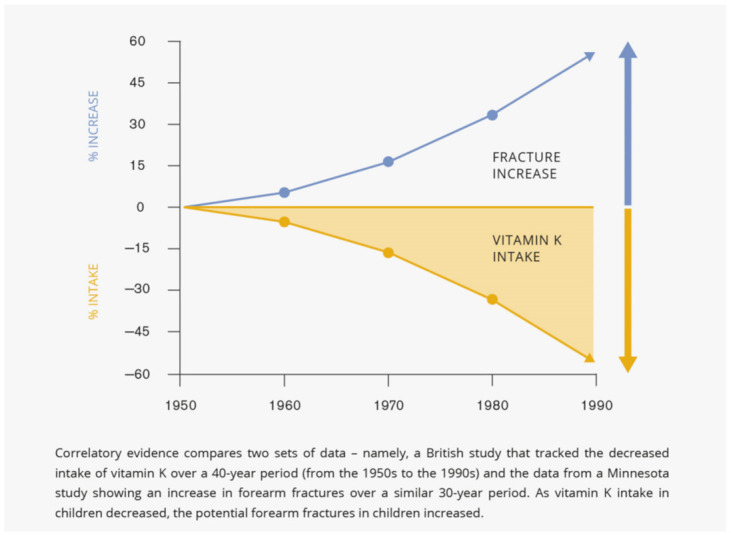
Low vitamin K intake corresponds with increased fracture risk.

Considerable noteworthy and correlatory evidence emerges from the comparison of the two sets of data—the decreased intake of vitamin K over a 40-year period (from the 1950s to the 1990s) and the data from Minnesota inquiry presenting an increase in broken forearms over a similar 30-year period—as vitamin K intake lessened in the youth population, the potential forearm fractures in children heightened (Figure 2).

The research also showed that children have the highest tissue-specific vitamin deficiency. Vitamin K status was most frequently evaluated by the percentage of active osteocalcin (cOC), vitamin K-dependent protein, important for bone.

When the vitamin K status of bone in healthy children was contrasted with that of adults for the needs of a cross-sectional study, a noticeable elevation of the ratio of uncaroboxylated (inactive) osteocalcin (ucOC) into carboxylated (active) osteocalcin was observed in children, thus indicating a poor vitamin K status. Moreover, in the children’s group the researchers also found a considerable correlation between bone markers for bone metabolism and ucOC and cOC. These deductions imply markedly low levels of vitamin K in the bone during growth [77].

A separate study found that the largest tissue-specific vitamin deficiency is common among children and adults above 40 years, and thus menaquinone-7 (MK-7) supplementation may have a beneficial influence on improving the extra-hepatic vitamin K status [78].

Much research demonstrates that children need more vitamin K than adults due to various health disorders and therapeutics used in pediatric practice. For instance, vitamin K deficiency often goes hand in hand with disorders of fat malabsorption. Shortage of vitamin K manifests itself with easy bruising and bleeding, and children suffering from malabsorption also have tendencies to poor bone health and osteoporosis [79,80]. Supplementation is a good solution in dealing with children’s vitamin K deficiencies.

## 6. Infants and K Deficiency

Low vitamin K status is much more common in neonates than adults, due to both endogenous and exogenous deficiency. The endogenous case has been associated with poor intestinal colonization by bacteria [81] and a defective vitamin K epoxide reductase (VKOR), which might lead to severe coagulopathy and/or skeletal defects [82]. Several common polymorphisms of VCOR have been detected within subunit 1 (VKORC1), which is essential for VKOR enzymatic activity, and have been found to influence vitamin K recycling in hepatic as well as extrahepatic tissues. Some polymorphisms of VKOR gene lead to decrease in VKOR enzyme in half [83].

The reasons for the exogenous insufficiency arise from limited vitamin K transport across the placental barrier and its low accumulation in breast milk.

The main exogenous source of vitamin K in newborns, which is almost exclusively milk, is not able to suitably equalize the insufficient endogenous production, because mother’s milk contains 1 to 4 mcg/L of vitamin K1 (and a much lower concentration of vitamin K2). Throughout the first 6 months of life the median amount of vitamin K that entirely breast-fed infants receive has been described to be below 1 mcg/day. Surprisingly, this amount in infants, who, as their meal get a typical supplemented formula, is approximately 100-fold higher. This argument should encourage breast-feeding mothers to seriously consider supplementation with vitamin K2 [79,84,85,86].

**Figure 3:**
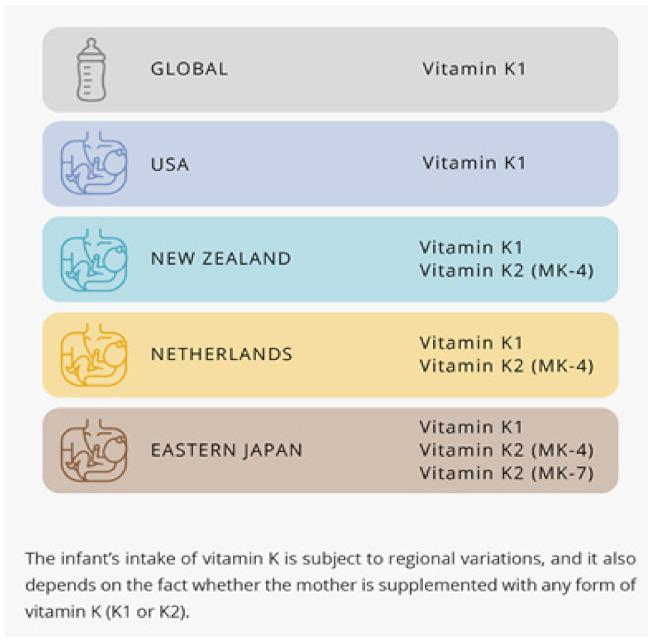
Presence of K1 and K2 from formula vs. breastmilk (regional differences).

The content of vitamin K in breast milk depends on the geographical region [87]. Researchers from the United States found only vitamin K1 to be present in human colostrum and milk when menaquinones were not detected [88]. Vitamins K1 as well as vitamin K2 (the form MK-4 and MK-7) were found in the breast milk of Japanese mothers. Japanese mothers living in eastern part of the country had a higher concentration of MK-7 in milk than mothers living in western Japan, probably due to differences in dietary foods [89,90]. However, increasing maternal intake of vitamin K with meaningful doses has been shown to increase the vitamin K content of human milk [91,92]. Moreover, it was found that dietary vitamin K1 is a source of vitamin K2 in breast milk [93] (Figure 3).

The second endogenous culprit of the neonates’ deficiency of vitamin K at birth is the poor transport of this nutrient across the placenta from mother to infant. Particularly in preterm pregnancies, little vitamin K actually crosses the placenta from mother to infant [94]. However, this could be attributed to insufficient maternal levels of vitamin K in the first place. In fact, several studies have demonstrated that supplementation with vitamin K in more advanced pregnancy increases plasma concentrations of this nutrient and enhances coagulation function of future mothers [92]. In addition, administration of vitamin K to pregnant women resulted in improved vitamin K-dependent coagulation factors in umbilical blood and reduced the incidence as well as the severity degree of periventricular-intraventricular hemorrhage (PIVH) in premature infants [95,96]. Furthermore, late-preterm babies, whose mothers were given vitamin K at imminent risk of preterm labor, were able to achieve a clotting status approaching that of full-term neonates and are less liable to develop PIVH [97].

## 7. VKDB Is More Common in Asia than Western Countries

Healthy newborn infants have a very fragile vitamin K coagulation status [98]. In the past vitamin-responsive bleeding used to be called hemorrhagic disease of the newborn (HDNB), but nowadays it is more informatively called vitamin K deficiency bleeding (VKDB) [99].

Based on epidemiological studies of the prevalence of late VKDB, researchers have demonstrated that China, Japan, countries within Southeast Asia, such as Thailand and Vietnam, and also Australia have greater rates of VKDB than in the remaining parts of the world.

Vitamin K insufficiency in Thai mothers (represented by detectable PIVKA-II levels) was due to reduced dietary K intakes during gestation period [100].

The Japanese nationwide surveys found that the 87.7% of VKDB cases reported involved exclusive breastfeeding. Guidelines for expectant mothers in Japan who use drugs that could impair the absorption of vitamin K (excluding warfarin) say that they should be provided regular doses of 15–30 mg vitamin K daily 2 to 4 weeks before delivery or their newborn should receive 0.5–1 mg IM vitamin K2 administration [26,101,102,103,104].

## 8. The Recommendations for Infant’s Intake

The infant’s intake of vitamin K is a subject to regional variations, and it also depends on the fact whether the mother is supplemented with any form of vitamin K (K1 or K2).

In the USA and Canada, the adequate intakes (AIs) for babies are based on the calculated average vitamin K1 intake of healthy breastfed infants and the expectation that newborns receive prophylactic vitamin K1 at delivery, as recommended by American and Canadian pediatric societies [92]. In US, the AI for infants aged 0–6 months is 2 μg/day [92].

The European Society for Paediatric Gastroenterology Hepatology and Nutrition (ESPGHAN) recommends as follows: healthy new-born infants should either receive 1 mg of vitamin K1 by intramuscular injection at birth; or 3 × 2 mg vitamin K1 orally at birth, at 4 to 6 days and at 4 to 6 weeks; or 2 mg vitamin K1 orally at birth, and a weekly dose of 1 mg orally for 3 months [105].

In Japan an AI of 4 mg/day for infants aged 0–5 months was determined by multiplying the average milk intake and the average vitamin K content of milk, and assuming the presence of the oral administration of vitamin K just after birth in clinical settings [106]. However, it is important to mention that, in Japan, the sum of the quantity of vitamin K1, MK-4, and MK-7 was employed in determining the daily recommended intakes (DRIs) for vitamin K.

## 9. Conclusions

Vitamin K2, as MK-7, has a documented history of safe and effective use in children, as well as in adults. When reading product labels, one can notice that MK-7 is already mentioned as a form of vitamin K. The absent side effects of MK-7 also precluded many authorities, such as The Food and Nutrition Board of the Institutes of Medicine (IOM), the European Commission, the Expert Group on Vitamins and Minerals in UK (UK EVM), and the World Health Organization (WHO)/Food and Agriculture Organization (FAO), from establishing a tolerable upper intake level for any form of vitamin K. This is because exceeding the adequate intake is safe, even when someone additionally ingests MK-7 from foods and supplements. The only possible contradiction is the use of anticoagulant drugs, such as coumarins, which may interfere with vitamin K cycle.

Vitamin K2, especially as MK-7, which lasts longer in the body than MK-4, has important roles to play in the health of children, including the performance of various physiological functions such as coagulation, promoting bone mineralization, and a healthy cardiovascular system. Furthermore, vitamin K-dependent matrix-gla protein (MGP) helps inhibit arterial calcification (which may begin in childhood), so early supplementation with vitamin K2 may contribute to good cardiovascular health in infancy, puberty, and beyond. The current adequate intake level of vitamin K for pregnant and nursing women is 90 mcg. Likewise, research suggests that 45–50 mcg/day MK-7 is an appropriate intake range for children [107,108,109].

The good news shared by such trustworthy bodies as the European Food Safety Authority (EFSA), the UK EVM, the IOM, and WHO, which was also supported by clinical and nonclinical data, clearly states that using MK-7 as a dietary supplement according to the recommended dosages is safe [110,111,112].

## Figures and Tables

**Table 1:** Distinguishing Between Vitamins K1 and K2.

	Vitamin K1 (Phylloquinone)	Vitamin K2 (Menaquinones)
**Major source**	Leafy greens, fruits, vegetable oils	Fermented food (natto), animal products (meat, dairy)
**Half time**	∼3 h	MK-4: ∼1.5 h MK-7: ∼70 h
**Efficacy**	Low. High dosages (mg) are needed to improve vitamin K status	MK-4: Low. High dosages (mg) are needed to improve vitamin K status MK-7: High. Low dosages (mcg) are needed to improve vitamin K status
**Mechanism of action**	Activation of VKDPs (mainly hepatic) Antioxidant Anti-inflammatory effects	Activation of VKDPs (hepatic and extra hepatic) Antioxidant Anti-inflammatory effects Transcriptional regulator Mitochondrial electron carrier, helping to maintain normal ATP levels
**Utilization**	Primarily used by liver, helps maintaining healthy blood clotting	Can be used by liver, helps maintaining healthy blood clotting Available for extra hepatic tissues. Essential for bone strength, blood vessel health, brain development and more

## Data Availability

Not applicable.
